# Prevalence and predictors of chronic kidney disease of undetermined causes (CKDu) in Western Kenya’s “sugar belt”: a cross-sectional study

**DOI:** 10.1186/s12882-023-03213-2

**Published:** 2023-06-06

**Authors:** Michelle H. Hathaway, Crystal L. Patil, Aloyce Odhiambo, Dickens Onyango, Samuel Dorevitch

**Affiliations:** 1 Division of Environmental and Occupational Health Sciences, School of Public Health, University of Illinois Chicago, 1603 W. Taylor St., Chicago, IL 60612 USA; 2 Department of Human Development Nursing Science, College of Nursing, University of Illinois Chicago, 845 S. Damen Ave., MC 802, Chicago, IL 60612 USA; 3 Safe Water and AIDS Project, Behind Royal City Garden Hotel, Milimani Estate, Off Aga Khan Road, P.O. Box, Kisumu, 3323-40100 Kenya; 4 County Department of Health, County Government of Kisumu, Kisumu, Kenya

**Keywords:** Chronic kidney disease of undetermined causes, HIV, Occupational health, Environmental epidemiology, Global health

## Abstract

**Background:**

Epidemics of chronic kidney disease of undetermined causes (CKDu) among young male agricultural workers have been observed in many tropical regions. Western Kenya has similar climatic and occupational characteristics as many of those areas. The study objectives were to characterize prevalence and predictors of CKDu, such as, HIV, a known cause of CKD, in a sugarcane growing region of Kenya; and to estimate prevalence of CKDu across occupational categories and evaluate if physically demanding work or sugarcane work are associated with reduced eGFR.

**Methods:**

The Disadvantaged Populations eGFR Epidemiology Study (DEGREE) protocol was followed in a cross-sectional study conducted in Kisumu County, Western Kenya. Multivariate logistic regression was performed to identify predictors of reduced eGFR.

**Results:**

Among 782 adults the prevalence of eGFR < 90 was 9.85%. Among the 612 participants without diabetes, hypertension, and heavy proteinuria the prevalence of eGFR < 90 was 8.99% (95%CI 6.8%, 11.5%) and 0.33% (95%CI 0.04%, 1.2%) had eGFR < 60. Among the 508 participants without known risk factors for reduced eGFR (including HIV), the prevalence of eGFR < 90 was 5.12% (95%CI 3.4%, 7.4%); none had eGFR < 60. Significant risk factors for reduced eGFR were sublocation, age, body mass index, and HIV. No association was found between reduced eGFR and work in the sugarcane industry, as a cane cutter, or in physically demanding occupations.

**Conclusion:**

CKDu is not a common public health problem in this population, and possibly this region. We recommend that future studies should consider HIV to be a known cause of reduced eGFR. Factors other than equatorial climate and work in agriculture may be important determinants of CKDu epidemics.

**Supplementary Information:**

The online version contains supplementary material available at 10.1186/s12882-023-03213-2.

## Background

In the 1990s, cases of end-stage renal disease of young, male agricultural workers with no history of hypertension, diabetes, or glomerular disease, were reported by clinicians in El Salvador [1]. Since then, an epidemic of chronic kidney disease of undetermined causes (CKDu) has been identified among mostly young men in agricultural areas of Central America [2–5]. Sometimes referred to as Mesoamerican nephropathy [6], chronic interstitial nephritis in agricultural communities (CINAC) [7, 8], Uddanam nephropathy, and Sri Lanka nephropathy, cases have also been described in Sri Lanka [9–13] and Andhra Pradesh, India [14–16].

Pesticides, informally produced alcohol, dietary sugar, heavy metals in drinking water, infectious agents, and nonsteroidal anti-inflammatory drugs [17–21], have been evaluated as etiologic factors, but none have been definitively established as cause(s) of CKDu. Presently, recurrent dehydration in the context of physically demanding work in hot climates is a focus of CKDu etiologic research [18, 22–28]. The hot climate of Western Kenya, as well as the widespread prevalence of manual labor in agriculture and other industries, are not unlike climatic conditions and occupational hazards in Central America, Sri Lanka, and India. Thus, if physically demanding work in a hot climate is a risk for CKDu, the condition may be found in Kenya, particularly in sugar growing regions. Only a few studies have addressed CKDu in sub-Saharan Africa. Ekiti et al. [29] described a 3.4% CKDu prevalence among sugarcane workers in Cameroon. A study conducted in Malawi utilized the Disadvantaged Populations eGFR Epidemiology (DEGREE) Study protocol, a standardized approach to evaluate CKDu [30, 31] and reported a 0.2% prevalence [32].

CKD is a common complication of human immunodeficiency virus (HIV) infection, affecting 3.5%–48.5% of people with HIV in sub-Saharan Africa [33–35]. In Western Kenya, among 373 HIV-infected, retrovirally-naive participants without diabetes or hypertension, 11.5% were found to have a creatinine clearance < 60 ml/min indicating possible CKD [36]. The prevalence of HIV infection among adult Kenyans is 4.9% and in Kisumu County it is 17.5% [37]. Thus, HIV should be considered in assessing the prevalence of CKDu in Kenya.

Numerous prevalence studies of CKDu have recruited participants from specific occupational groups, rather than being population based. Associations between CKDu and occupation have been addressed in population-based studies, though the methods for defining and categorizing occupation has varied [5, 27, 38]. The use of standardized methods of occupational classification should help advance our understanding of work-related risk factors for CKDu.

We utilized the DEGREE protocol to characterize the prevalence and risk factors for CKDu in Kisumu County, a sugar-growing region of Western Kenya. The DEGREE definition of CKDu excludes cases with diabetes, hypertension, or heavy proteinuria. Given that HIV accounts for some of the CKD cases, we evaluated renal function among those with and without HIV. Additionally, we used a standardized job classification system [39] and an empirically-based categorization of physical demand at work [40] to evaluate associations between occupation and CKDu.

## Methods

### Study design and study setting

**Fig. 1:**
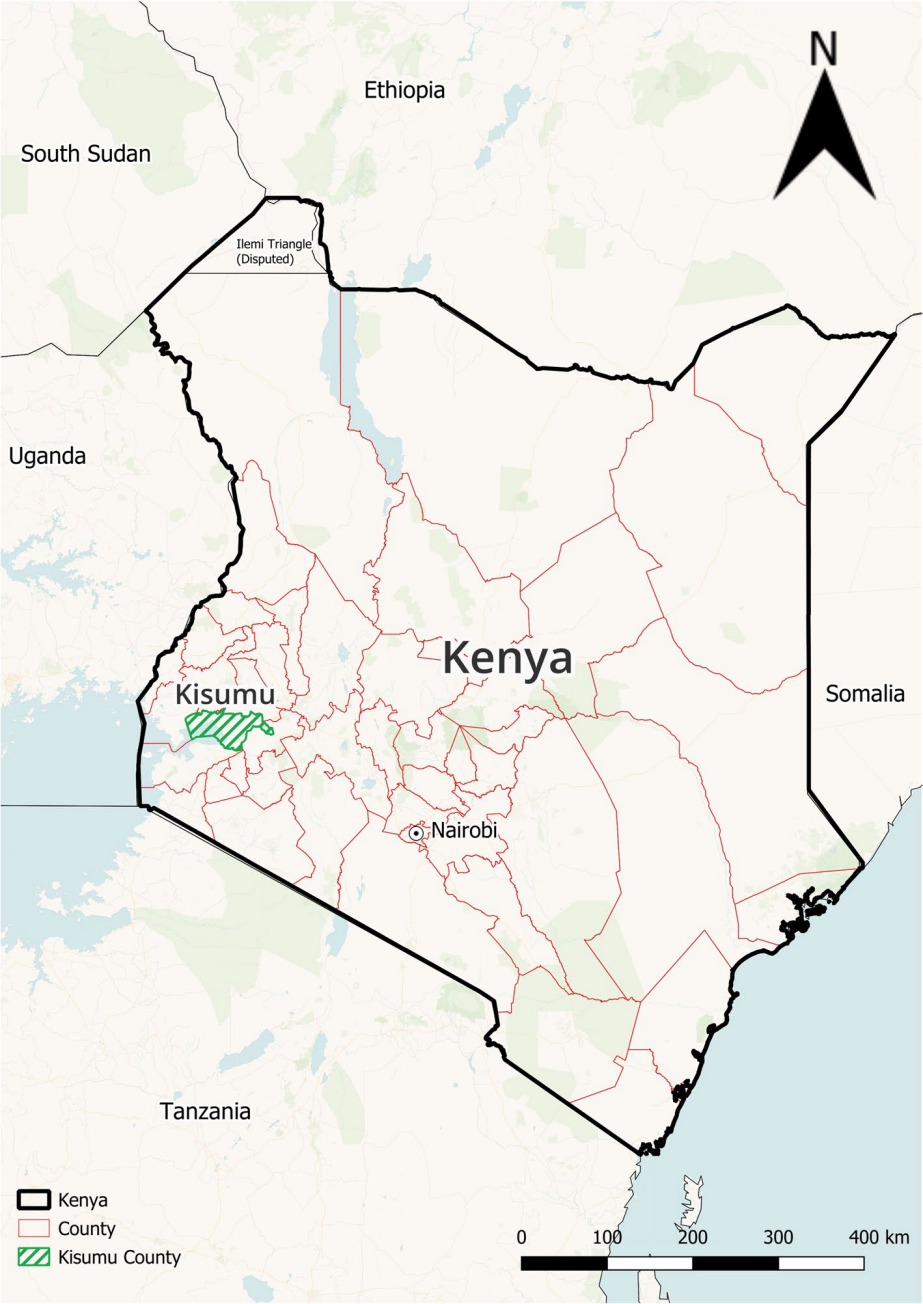
Kisumu County, Kenya

A cross-sectional, population-based household survey was completed following the DEGREE protocol [30, 31] to allow for regional and international comparisons of CKDu prevalence. The study took place in Western Kenya’s “sugar belt” in a rural area of the Muhoroni Sub-County (population: 154,116, land area: 657.5 km^2^, population density: 234 persons/km^2^) located within Kisumu County (Fig. 1). Within Muhoroni Sub-County, three sublocations – Owaga, Muhoroni East and Tonde, which represent the catchment area of Muhoroni County Hospital (the local government hospital), and each consist of ten villages – comprised the sampling frame. Owaga (Latitude: -0.153378, Longitude: 35.221355) and Tonde (Latitude: -0.130709, Longitude: 35.198380) are rural while Muhoroni East (Latitude: -0.145457, Longitude: 35.206700) includes a more urbanized town center.

The DEGREE protocol calls for an enrollment goal of 1,000 so that if the true prevalence of eGFR < 90 ml/min is 5%, the resulting 95% confidence interval would be 3.6%-6.4% [30]. Four villages were randomly selected from each of the sublocations, totaling twelve villages and 1,268 households. Recruitment and enrollment were at the household level with a household defined as a structure in which the family sleeps at night. All the households from each village were approached for recruitment of one individual per household. Male and non-pregnant female adults between 18 to 59 years who resided in one of the three sublocations for at least six months prior to enrollment were eligible to participate. To ensure that men and women were equally represented in the sample, the sex of the participant to be targeted for enrollment at each household was predetermined by randomization.

### Participant recruitment

Between 27^th^ July and 30^th^ November in 2020, five pairs of research staff, each pair consisting of an interviewer and a licensed phlebotomist, went house to house in the daytime and early evenings to recruit participants Monday through Friday. Additionally, teams recruited participants on one weekend per sublocation. The questionnaire was administered by the interviewer in *Dholuo* or *Kiswahili* (based on the participant preference), and responses were entered directly into password-protected electronic tablet devices using SurveyCTO software. The questionnaire included the DEGREE core protocol questionnaire and the DEGREE optional renal protocol (e.g., previous disease diagnoses and medications). Questions regarding occupational history, health habits, and drinking water source originated from a community-based CKDu study [41].

### Anthropometry and health metrics

Height and weight were measured in duplicate (and in triplicate if the difference between the first two measurements was more than 0.5 cm or 0.2 kg) using the Road Rod Portable Stadiometer (Hopkins Medical Products) and four calibrated Seca digital floor scales (Seca 813) and one calibrated Tanita digital floor scale (HD-314). Blood pressure was measured with a digital Upper Arm Blood Pressure Monitor (Omron model BP7350) taken three times, five minutes apart while the participant was seated.

During the household visit, a 5 mL venous blood sample was collected in a red top vacutainer (BD) and a 50 mL urine sample was collected. Samples were transported in coolers with ice packs to the Muhoroni County Hospital laboratory where urine samples were analyzed within 20 min of arrival using the Optical Clarity Urocheck 120 Urine Analyzer. Serum tubes were centrifuged for 10 min at 2400 revolutions per minute (RPM) and then aliquoted into 1.8 mL microcentrifuge tubes (Jiangsu Kangjie Medical Devices Co. Ltd) and frozen at -20 °C in a freezer with a backup generator. Frozen serum samples were transported on ice approximately once per week to the Kenya Medical Research Institute (KEMRI) HIV-R Laboratory in Kisumu City for analysis. There, creatinine and cystatin C were measured using the Roche Cobas Integra 400 plus. The instrument was calibrated for creatinine analysis using isotope dilution mass spectrometry reference standards.

### Reduced eGFR and CKDu: definitions

The DEGREE definition of CKDu is an eGFR of < 60 mL/min/1.73m^2^ based on a single serum creatinine measurement in individuals without diabetes, hypertension, or heavy proteinuria [30, 31]. We followed the convention of prior studies that utilized the DEGREE protocol and also assessed eGFR < 90 as an endpoint because of low observed prevalence of eGFR < 60 [32, 42]. Table 1 defines the terms used for renal function categories. The DEGREE definition excludes those with diabetes, hypertension, heavy proteinuria, or underlying kidney disease. The DEGREE-Kenya definition also excludes those diagnosed with HIV. Diabetes was defined by a self-report of physician-diagnosed diabetes or taking medication for diabetes. Hypertension was defined as systolic blood pressure ≥ 140 mmHg or diastolic blood pressure ≥ 90 mmHg and/or a self-report of taking medication for hypertension. Heavy proteinuria was defined as a urine dipstick of ≥ 2 + (approximately equivalent to albumin-to-creatinine ratio > 300 mg/g). HIV status was determined by asking whether the participant has ever been told they have one of six listed health conditions, one being HIV.

**Table 1:** Definitions for kidney function categories with and without risk factors and heavy proteinuria

**eGFR categories (ml/min/1.73m**^**2**^**)**	**Participant subsets**		
	**All participants**	**Subset 1: no DM, HTN, or heavy proteinuria**	**Subset 2: no DM, HTN, heavy proteinuria, or HIV**
≥ 90 (normal kidney function)	Normal eGFR	Normal eGFR_DEGREE_	Normal eGFR_Kenya_
≥ 60 to < 90 (mild kidney dysfunction)	Reduced eGFR	Reduced eGFR_DEGREE_	Reduced eGFR_Kenya_
< 60 (moderate or established kidney dysfunction)	Proxy of CKD^a^	CKDu_DEGREE_	CKDu_Kenya_

^a^No case would be considered CKD because serum creatinine was measured only on one occasion

### Occupation and physical demands of work

Participants were asked about their current main occupation, and they described their job duties. Based on the participants’ responses, occupation was coded using the International Standard Classification of Occupations 2008 (ISCO-08) [39]. ISCO-08 coding includes the Major Group level (broadest, 1-digit code), Sub-Major Group (2-digit), Minor Group (3-digit), or Unit Group (most specific, 4-digit). Participants who were unemployed, a student, a homemaker, or unclassifiable – which are not ISCO-08 categories – were assigned codes accordingly. Classification was done independently by two coders; discrepancies in coding were adjudicated by consensus. ISCO-08 Major Groups were classified by occupational physical intensity into low-, moderate-, and high-intensity groups using a method validated through the use of energy expenditure and metabolic equivalents metrics [40]. In addition to using the ISCO-08, sugarcane cutting was examined separately for associations with low eGFR because of the association found with CKDu in earlier studies. A “current sugarcane cutter” was defined as someone who reported currently working as a manual sugarcane cutter for at least three consecutive months and a “former sugarcane cutter” was someone who reported having previously cut sugarcane for at least three consecutive months during the past ten years but not working as a cutter at the time of enrollment.

### Statistical analysis

Normality, central tendency, and variability were summarized for continuous variables, and frequencies were summarized for categorical variables. eGFR was calculated using the Chronic Kidney Disease – Epidemiology Collaboration (CKD-EPI) equation based on serum creatinine and race was not used in the equation [43–45].

Logistic regression was used to identify potential associations between predictor variables and eGFR < 90 mL/min/1.73m^2^ (which combines the mild- and established kidney dysfunction categories). Predictors of eGFR < 90 mL/min/1.73m^2^ that were significant with a *p*-value of < 0.15 were added to multivariate logistic regression models (*p*-value < 0.05 with 95% confidence intervals). Based on a priori research questions about occupation, sugarcane work, and occupational physical exertion, these terms were forced into models, regardless of their statistical significance as predictors of eGFR < 90 and a backward selection process was used to arrive at a final multivariate model. All analyses were conducted using SAS version 9.4 (SAS Institute, Cary, NC).

### Human subjects research protections

The University of Illinois Chicago (IRB Protocol #: 2019–1236) and Maseno University Ethics Review Committee in Kisumu, Kenya (IRB Protocol #: MSU/DRPI/MUERC/00798/19) reviewed and approved study protocols. Participants provided written informed consent in their preferred language.

## Results

### Participant enrollment and demographic characteristics

**Fig. 2:**
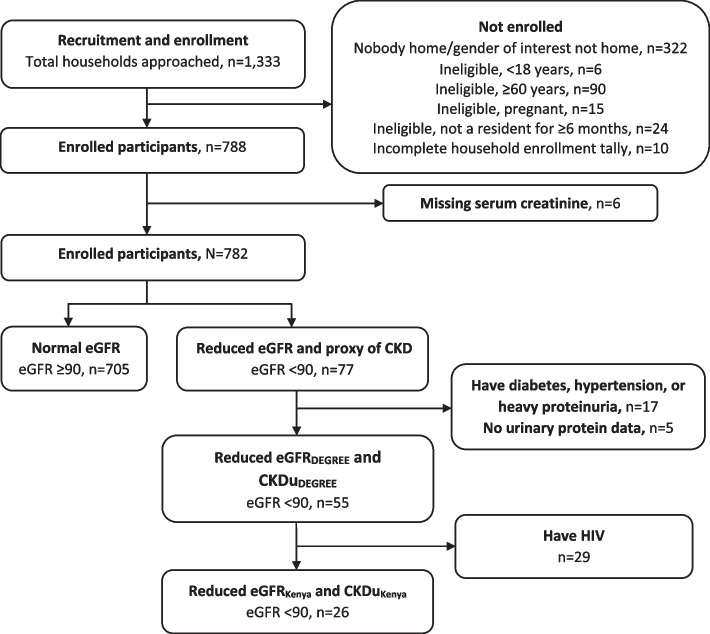
Participant enrollment and classification by eGFR categories

Participant enrollment is summarized in Fig. 2. The 322 “nobody home” households include those in which nobody was home and those in which nobody of the gender to be enrolled (determined in advance) was at home.

Table 2 summarizes the sociodemographics, health habits, and anthropometrics of study participants. Of the 782 individuals for whom serum creatinine data were available the median (5^th^, 95^th^ percentile) age was 35.0 years (20.0, 56.0), the median BMI was 22.8 kg/m^2^ (18.3, 33.1), the median systolic blood pressure was 120.2 (101.7, 148.7) and the median diastolic blood pressure was 77.0 (63.3, 97.0). Twelve participants (1.5%) met the definition of a current sugarcane cutter for at least three consecutive months. Overall, 84 (10.7%) participants have ever cut sugarcane in their lifetime for income or have done a job related to sugarcane. One-hundred and twenty-five (16.0%) participants self-reported having HIV.

**Table 2:** Sociodemographics and anthropometrics of study participants, *n* = 782

Variable	n	%
**Sex**		
Female	392	50.1
Male	390	49.9
**Sublocation**		
Tonde	233	29.8
Muhoroni East	260	33.2
Owaga	289	37.0
**Age**		
18 – 29 years	243	31.1
30 – 39 years	252	32.2
40 – 49 years	170	21.7
50 – 59 years	117	15.0
**Level of education**		
Did not attend school	10	1.3
Some primary school	175	22.4
Completed primary school	207	26.5
Some secondary school	117	15.0
Completed secondary school	158	20.2
Some undergraduate school	80	10.2
Completed undergraduate school	29	3.7
Postgraduate education	6	0.8
**Body Mass Index (BMI) (kg/m**^**2**^**)**		
Underweight (≤ 18.5)	46	5.9
Normal (> 18.5 to ≤ 25)	475	60.7
Overweight (> 25 to ≤ 30)	178	22.8
Obese (> 30)	83	10.6
**Health habits**		
Smoker and/or alcohol drinker	167	21.4
Non-smoker and non-alcohol drinker	615	78.6
**Self-reported HIV**		
Yes	125	16.0
No	657	84.0
**Blood pressure and antihypertensive medications**		
≥ 140 or ≥ 90 or on antihypertensive medications	111	14.2
< 140 and < 90 and not on antihypertensive medications	671	85.8
**Physician diagnosed diabetes or on medication for diabetes**		
Yes	8	1.0
No	774	99.0
**Proteinuria**		
Negative, Trace or 1 +	717	91.7
2 + or 3 +	5	0.6
Missing	60	7.7

### eGFR and prevalence of eGFR** < **90 with and without known causes of CKD

**Fig. 3:**
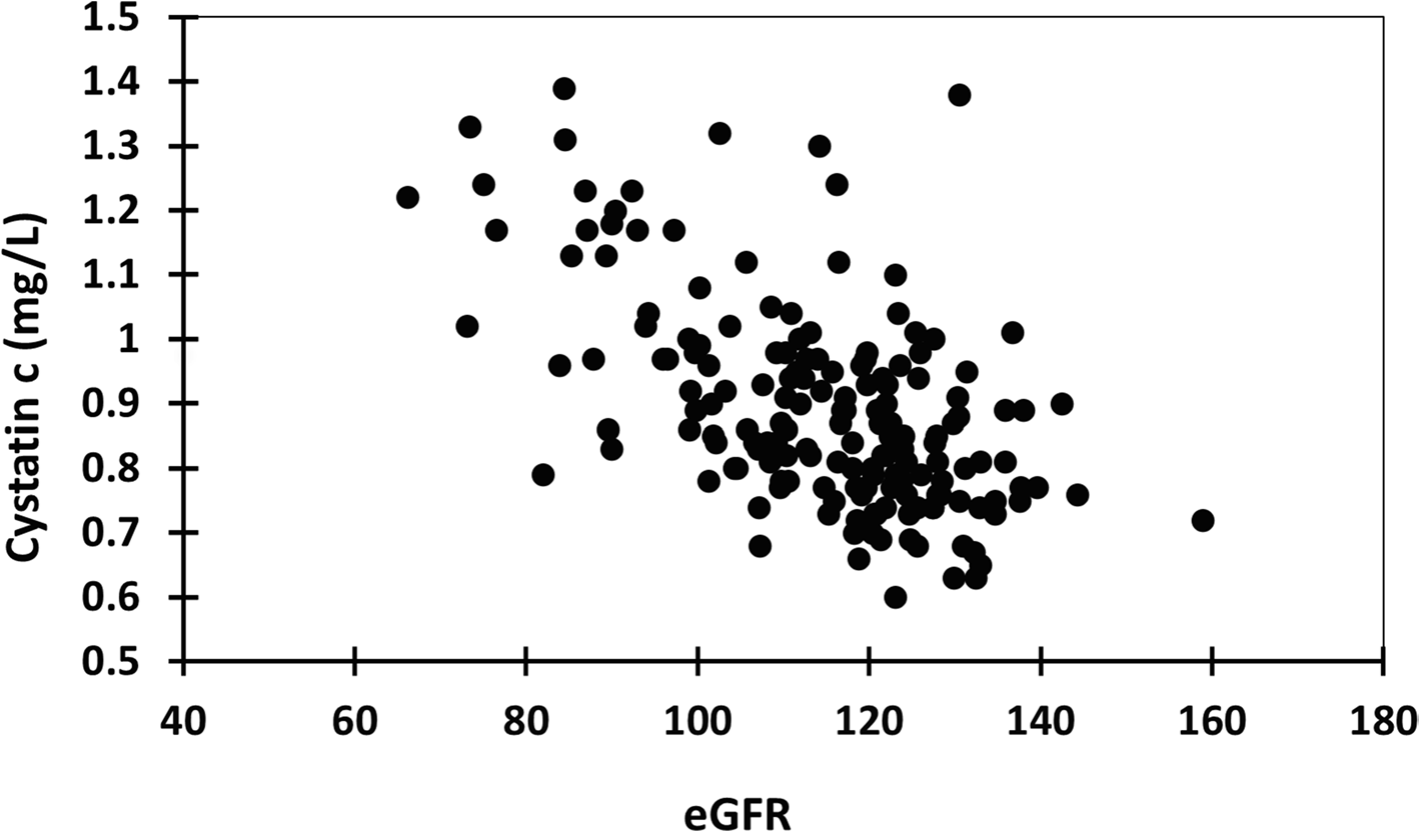
Scatterplot of eGFR and Cystatin c correlation

The median (5^th^, 95^th^ percentile) serum creatinine was 0.71 (0.49, 1.02) mg/dL and the median eGFR was 115.3 (82.2, 137.6) mL/min/1.73m^2^. The prevalence of eGFR < 90 was 9.85%. Cystatin c data were available for 173 participants and completed on a random subset of samples throughout the data collection phase. A single cystatin c testing kit had 225 tests, including those used for controls. The median (5^th^, 95^th^ percentile) of cystatin c was 0.86 (0.68, 1.23) mg/L. eGFR and cystatin c were moderately correlated (*r* = -0.52, Spearman *p*-value < 0.0001) (Fig. 3). Among Subset 1 participants (*n* = 612), the median (5^th^, 95^th^ percentile) eGFR was 117.0 (83.7, 137.7) mL/min/1.73m^2^ and the prevalence of eGFR < 90 was 8.99% (95%CI 6.8%, 11.5%) (*n* = 55) with 0.33% (95%CI 0.04%, 1.2%) (*n* = 2) having an eGFR < 60. Among Subset 2 participants (*n* = 508), the observed prevalence of eGFR < 90 was 5.12% (95%CI 3.4%, 7.4%) (*n* = 26); none had an eGFR < 60.

### Occupational and physical intensity classifications and prevalence of reduced eGFR

Table 3 displays the distribution of overall median eGFR (5^th^, 95^th^ percentile) by ISCO-08 Major Groups 1–9 and other work status categories (e.g., homemaker). Skilled Agricultural, Forestry and Fishery Workers and Elementary Occupations, which includes agriculture and non-agriculture manual jobs, together accounted for 41.6% of participants. The prevalence of eGFR < 90 was about 12% across the major groups and other work status categories except for Major Group 1: Managers, who had a 33.3% prevalence. Among agricultural workers, which included almost 35% of all participants, the prevalence of eGFR < 90 was 12.5% (*n* = 34). Among those without diabetes, hypertension, and heavy proteinuria, the prevalence of reduced eGFR_DEGREE_ was 12.9% (*n* = 27) and 8.6% (*n* = 14) for reduced eGFR_Kenya_. Among participants with an ISCO-08 classification and eGFR (*n* = 604), there was no statistically significant association between the high-intensity group and an eGFR < 90 (OR: 1.29 [95%CI 0.75, 2.22], *p* = 0.36). Among Subset 1 participants there was a suggestion of an association between eGFR < 90 and the high-intensity group (OR: 1.73 [95%CI 0.87, 3.4], *p* = 0.12), however, it was not statistically significant. Lastly, an association between the high-intensity group and an eGFR < 90 was not present for reduced eGFR_Kenya_ (*p* = 0.48).

**Table 3:** Distribution of eGFR by ISCO-08 category and occupational physical intensity group for all participants

	**eGFR (** ***n*** ** = 782)**				**eGFR categories, n (%)**	
	**n (%)**	**Median**	**5th Pctl**	**95th Pctl**	** < 90, *n* = 77**	** ≥ 90, *n* = 705**
**ISCO-08 Major Groups 1–9**						
Major Group 1: Managers	6 (0.8)	102.9	84.1	125.9	2 (33.3)	4 (66.7)
Major Group 2: Professionals	47 (6.0)	111.8	87	135.7	3 (6.4)	44 (93.6)
Major Group 3: Technicians and Associate Professionals	18 (2.3)	112.8	62.6	143.6	2 (11.1)	16 (88.9)
Major Group 4: Clerical Support Workers	2 (0.3)	112.5	106.9	118	0 (0)	2 (100)
Major Group 5: Services and Sales Workers	120 (15.4)	118.1	81.4	135.3	11 (9.2)	109 (90.8)
Major Group 6: Skilled Agricultural, Forestry and Fishery Workers	163 (20.8)	109	79.6	132.3	20 (12.3)	143 (87.7)
Major Group 7: Craft and Related Trades Workers	64 (8.2)	110.8	85.5	132.4	7 (10.9)	57 (89.1)
Major Group 8: Plant and Machine Operators and Assemblers	21 (2.7)	110.3	80.5	130.4	3 (14.3)	18 (85.7)
Major Group 9: Elementary Occupations	163 (20.8)	115.8	82.2	132.9	21 (12.9)	142 (87.1)
Total ISCO-08 Major Groups	604 (77.2)	113.0	81.1	133.9	69 (11.4)	535 (88.6)
**Other work status categories**						
Student	53 (6.8)	130.4	105.8	158.8	0 (0)	53 (100)
Homemaker	40 (5.1)	113	73.6	144.2	6 (15.0)	34 (85.0)
Unemployed	63 (8.1)	123.4	94.8	138.6	1 (1.6)	62 (98.4)
Not classifiable	22 (2.8)	119.9	93.1	136.7	1 (4.6)	21 (95.4)
**Occupational physical intensity group**						
Low-intensity occupational group	55 (7.0)	111.6	84.9	135.7	5 (9.1)	50 (90.9)
Moderate-intensity occupational group	159 (20.3)	115.8	76.3	136.2	16 (10.1)	143 (89.9)
High-intensity occupational group	390 (49.9)	112.1	81.1	132.8	48 (12.3)	342 (87.7)
Not classifiable	178 (22.8)	124.7	90.3	143.8	8 (4.5)	170 (95.5)

Of the ten sugarcane occupation definitions evaluated, we found that only work in the sugarcane industry (but not work as a current or former sugarcane cutter) was, in unadjusted models associated with reduced eGFR (OR: 2.84 [95%CI 1.18, 6.82], p: 0.02), reduced eGFR_DEGREE_ (OR: 2.99 [95%CI 1.07, 8.41], p = 0.04), and reduced eGFR_Kenya_ (OR: 5.59 [95%CI 1.46, 21.40], *p* = 0.01). However, none of these associations approached statistical significance in multivariate models.

### Risk factors for reduced eGFR

Table 4 displays the bivariate predictors associated with low eGFR (p < 0.15 level of significance, which were then entered into multivariate regression models). Three categories of low eGFR were evaluated: reduced eGFR, reduced eGFR_DEGREE_ (eGFR < 90 in the absence of diabetes, hypertension, or heavy proteinuria), and reduced eGFR_Kenya_ (which added to the DEGREE definition the exclusion of those with HIV). Covariates that, after model selection were found to be significant predictors (*p* < 0.05) in multivariate models, are shown in Table 5. The association that remained significant in models of reduced eGFR and reduced eGFR_DEGREE_, was sublocation. The odds of having eGFR < 90 was nearly three times greater among those living in Owaga or Muhoroni East sublocations relative to those living in Tonde after adjustment for covariates. The age distribution and prevalence of BMI, diabetes, hypertension, heavy proteinuria, and HIV were comparable in Tonde relative to the other two sublocations.

**Table 4:** Bivariate logistic regression of characteristics associated with reduced eGFR, reduced eGFR_DEGREE_, and reduced eGFR_Kenya_

**Variable**	**Reduced eGFR**			**Reduced eGFR**_**DEGREE**_			**Reduced eGFR**_**Kenya**_		
	**n**	**OR (95%CI)**	***p*** **-value**	**n**	**OR (95%CI)**	***p*** **-value**	**n**	**OR (95%CI)**	***p*** **-value**
**Sex**									
Female	392	1.64 (1.01, 2.65)	0.04	312	1.77 (1.00, 3.14)	0.05	244	2.12 (0.93, 4.85)	0.07
Male	390	Ref		300	Ref		264	Ref	
**Sublocation**									
Muhoroni East and Owaga	549	2.23 (1.21, 4.14)	0.01	414	2.02 (1.02, 4.01)	0.04	341	2.12 (0.78, 5.74)	0.13
Tonde	233	Ref		198	Ref		167	Ref	
**Age**									
Per 1-year increase	782	1.11 (1.08, 1.14)	< .0001	612	1.12 (1.09, 1.16)	< .0001	508	1.14 (1.09, 1.19)	< .0001
**Body Mass Index (BMI) (kg/m2)**									
Underweight (≤ 18.5), overweight (> 25 to ≤ 30), or obese (> 30)	307	2.11 (1.31, 3.40)	0.002	222	2.48 (1.41, 4.34)	0.0015	187	5.07 (2.09, 12.31)	0.0003
Normal (> 18.5 to ≤ 25)	475	Ref		390	Ref		321	Ref	
**Blood pressure**									
≥ 140 or ≥ 90 or on hypertensive medication	111	1.54 (0.84, 2.81)	0.16	-	-	-	-	-	-
< 140 and < 90 and not on hypertensive medication	671	Ref		-	Ref	-	-	Ref	-
**Previous diabetes diagnosis by a physician**									
Yes	8	5.68 (1.33, 24.23)	0.01	-	-	-	-	-	-
No	774	Ref		-	Ref	-	-	Ref	-
**Self-reported HIV**									
Yes	125	4.68 (2.83, 7.74)	< .0001	104	7.17 (4.00, 12.84)	< .0001	-	-	-
No	657	Ref		508	Ref		-	Ref	-
**Medication against HIV**									
Yes	122	4.25 (2.56, 7.06)	< .0001	102	6.18 (3.46, 11.07)	< .0001	-	-	-
No	659	Ref		509	Ref		-	Ref	-
Missing	1			1			-	-	-
**Tuberculosis**									
Yes	30	1.89 (0.70, 5.09)	0.20	25	2.69 (0.97, 7.46)	0.05	11	^a^	
No	752	Ref		587	Ref		497	Ref	
**Prescribed medications**									
Yes	248	3.48 (2.15, 5.63)	< .0001	189	3.86 (2.18, 6.82)	< .0001	89	0.84 (0.28, 2.52)	0.76
No	534	Ref		423	Ref		419	Ref	
**Antibiotic medication for infection**									
Yes	466	1.46 (0.89, 2.41)	0.13	364	1.58 (0.87, 2.88)	0.13	287	1.78 (0.76, 4.17)	0.18
No	316	Ref		248	Ref		221	Ref	
**Painkillers most days**									
Yes	347	1.24 (0.78, 2.00)	0.35	271	1.45 (0.83, 2.53)	0.18	221	2.16 (0.96, 4.86)	0.06
No	435	Ref		341	Ref		287	Ref	
**Herbal or traditional remedies**									
Yes	269	0.85 (0.51, 1.41)	0.53	212	0.62 (0.33, 1.16)	0.13	191	0.72 (0.31, 1.70)	0.46
No	513	Ref		400	Ref		317	Ref	
**Consumption of sugary drinks in past 7 days**									
5 or more drinks	57	2.09 (1.01, 4.33)	0.04	44	2.05 (0.87, 4.85)	0.10	32	1.25 (0.28, 5.56)	0.76
None to 4 drinks	725	Ref		568	Ref		476	Ref	
**Currently smoke tobacco**									
Yes	39	0.23 (0.03, 1.70)	0.15	33	0.30 (0.04, 2.26)	0.24	28	^a^	
No	743	Ref		579	Ref		480	Ref	
**Occupational physical intensity group**									
High-intensity	390	1.29 (0.75, 2.22)	0.36	298	1.73 (0.87, 3.41)	0.12	231	1.39 (0.55, 3.47)	0.48
Low- and moderate-intensity	214	Ref		158	Ref		138	Ref	
Missing/Unclassifiable	178			156			139		

^a^Quasi-complete separation of data points detected. The odds ratio (OR) could not be calculated

Reduced kidney function was defined as eGFR < 90 mL/min/1.73m^2^

*Ref* reference category for odds ratio (OR) calculation

**Table 5:** Multivariate logistic regression (adjusted full model) of characteristics associated with reduced eGFR, reduced eGFR_DEGREE_, and reduced eGFR_Kenya_

**Variable**	**Reduced eGFR**			**Reduced eGFR**_**DEGREE**_			**Reduced eGFR**_**Kenya**_		
	**n**^a^	**OR (95%CI)**	***p*** **-value**	**n**^a^	**OR (95%CI)**	***p*** **-value**	**n**	**OR (95%CI)**	***p*** **-value**
**Sublocation**									
Muhoroni East and Owaga	548	2.97 (1.52, 5.83)	0.002	413	2.95 (1.34, 6.50)	0.007	-	-	-
Tonde	233	Ref		198	Ref		-	Ref	-
**Age**									
Per 1-year increase	781	1.10 (1.07, 1.14)	< .0001	611	1.12 (1.08, 1.16)	< .0001	508	1.14 (1.09, 1.19)	< .0001
**Body Mass Index (BMI) (kg/m**^**2**^**)**									
Underweight (≤ 18.5), overweight (> 25 to ≤ 30), or obese (> 30)	307	1.84 (1.08, 3.13)	0.024	222	2.44 (1.26, 4.72)	0.008	187	4.26 (1.65, 10.97)	0.003
Normal BMI (> 18.5 to ≤ 25)	474	Ref		389	Ref		321	Ref	
**Self-reported HIV**									
Yes	125	4.11 (2.36, 7.15)	< .0001	104	6.05 (3.12, 11.73)	< .0001	-	-	-
No	656	Ref		507	Ref		-	Ref	-

^a^The total n for reduced eGFR and reduced eGFR_DEGREE_ is missing 1 observation because of a missing response to taking HIV medication

*Ref* reference category for odds ratio (OR) calculation

## Discussion

This population-based study in an agricultural region of Kenya found that chronic kidney disease of unknown origin was uncommon. Among the 782 participants with eGFR data, 9.85% had eGFR < 90, and of that, 0.51% had an eGFR < 60. However, among the 508 participants without DM, HTN, heavy proteinuria, or HIV, the observed prevalence of eGFR < 90 was 5.12% (95%CI 3.4%, 7.4%); none had an eGFR < 60. Self-reported HIV was common (16.0% of participants, which is similar to the reported 17.5% prevalence for Kisumu County) [37] and after adjusting for covariates, the odds (95%CI) of having eGFR < 90 among those with HIV was 4.11 (2.36, 7.15) times greater than among those without HIV. Prior studies have reported a high prevalence of CKDu among workers who engage in physically demanding work in hot climates, including sugarcane workers [2, 5, 17, 27, 46]. We found no association between physically demanding work and reduced eGFR.

The significant risk factors identified in the multivariate model associated with reduced eGFR and reduced eGFR_DEGREE_ were sublocation, increasing age, having a non-normal BMI, and HIV positivity. Increasing age and a non-normal BMI were significant predictors of reduced eGFR_Kenya_. Table 6 summarizes other study findings that utilized DEGREE methods [32, 42, 47, 48]. The estimated prevalence of reduced eGFR_DEGREE_ appears to be greater in Sri Lanka and the Southern region of India compared to Peru, Malawi, and the present study [32, 42, 47, 48]. This suggests that local factors not present in Kenya, Malawi, or Peru may be present in locations with epidemics of CKDu.

**Table 6:** Comparison of reduced eGFR, reduced eGFR_DEGREE_, and CKDu_DEGREE_ prevalence and risk factors across DEGREE protocol studies

First author and country/region	Total sample size	Reduced eGFR < 90	Proxy for CKD eGFR < 60	Reduced eGFR_DEGREE_ < 90	CKDu_DEGREE_ eGFR < 60	Associated risk factors with eGFR < 90 and < 60
Hamilton, et al. (2020). Malawi, southeast Africa	821	Did not report	Did not report	4.6% (95%CI 3.2, 6.3); *n* = 38	0.2% (95%CI 0.1, 0.9); *n* = 2	Increasing age, BMI
Ruiz-Alejos, et al. (2021). Northern Peru, Tumbes region	1,514 total sample; 1,272 after excluding those with DM, HTN, and HP	eGFR ≥ 60 to < 90: 16.6% (95%CI 14.8, 18.6)	1.7% (95%CI 1.1, 2.5); *n* = 26	eGFR ≥ 60 to < 90: 13%; *n* = 165	0.9% (95%CI 0.4, 1.5); *n* = 11	Low physical activity levels, kidney stones; sugarcane work was protective
Ruwanpathirana, et al. (2019). Anuradhapura, Sri Lanka	4,803 total sample; 3,351 after excluding those with DM, HTN, and HP	Did not report	12%; *n* = 576	Did not report	6.0% (95%CI 5.2, 6.8); *n* = 202	Advanced age, history of CKD among parents or siblings, living in areas classified as moderate and high CKDu-endemicity; agricultural work was not significant
O’Callaghan-Gordo, et al. (2019). Northern and Southern India^a^	12,500	Did not report	Did not report	eGFR ≥ 60 to < 90: 17.0% (95%CI 16.0, 17.0); *n* = 2,125	Overall: 1.6% (95%CI 1.4, 1.9). Varied from 1.4% in northern urban areas to 4.8% southern rural	Older age, residence in a rural area, being male, and less formal education for each 5 years of school
Western Kenya, Muhoroni Sub-County	782	9.85% (95%CI 7.9, 12.2); *n* = 77	0.51% (95%CI 0.14, 1.3); *n* = 4	8.99% (95%CI 6.8, 11.5); *n* = 55	0.33% (95%CI 0.04, 1.2); *n* = 2	Sublocation, increasing age, non-normal BMI, HIV positivity

*DM* diabetes mellitus, *HTN* hypertension, *HP* heavy proteinuria, *BMI* Body Mass Index, *HIV* Human Immunodeficiency Virus

^a^Data collected prospectively for other purposes but analyzed per DEGREE protocol

We observed that among participants residing in Owaga or Muhoroni East, after adjustment for confounders, the odds of having eGFR < 90 was three times higher than among those living in Tonde. The two sublocations at elevated risk differ from one another in terms of urbanization. Owaga is rural, like the low-risk sublocation of Tonde, while Muhoroni East is more urban. An explanation for this association is not obvious, as the age distribution and prevalence of BMI, diabetes, hypertension, and HIV were similar across the sublocations. Possible explanations might include contaminants in local drinking water sources and the use of fertilizers and pesticides on crops, though data needed to investigate those possibilities are not available. Focused investigations into the apparent sublocation clustering of cases are warranted to further evaluate environmental risk factors.

It is well-established that HIV is associated with impaired renal function [34–36, 49, 50]. We found that positive HIV status was strongly associated with reduced renal function. In single-predictor models, reported use of HIV medication was predictive of reduced eGFR and reduced eGFR_DEGREE_ categories, however, this association did not remain significant in the final multivariate model whereas HIV status did. Prior studies of CKDu have not addressed HIV or antiretroviral therapy (ART) medications as known causes of kidney dysfunction. Future studies of CKDu should evaluate HIV and ART medication history among participants and consider low eGFR among those with HIV to be of known cause. Consideration should be given to the standardization of asking sensitive questions like HIV status and collecting ART medication history to determine nephrotoxicity when in regions with high rates of HIV, and/or by including serologic testing for HIV in the study protocol. It is also possible that HIV accounted for some of the cases of CKDu in prior studies that did not address this known cause of impaired renal function.

The observed lack of association between occupational factors and reduced eGFR and CKDu is somewhat surprising. Studies conducted in Central America have found that workers who engage in physically demanding work in high temperatures, such as, sugarcane cutting, corn production, mining, and brickmaking, had a CKDu prevalence as high as 14.0% in Nicaragua and almost 19.0% in El Salvador [2, 5]. In our study participants who did sugarcane-related work but not specifically cane cutting, were more likely to have eGFR < 90 in unadjusted models. This association disappeared when evaluated in multivariate models. When we estimated the significance between eGFR < 90 and sugarcane cutting as an occupation (including both current and former cane cutters), we observed no association. This could mean that methods for cane cutting, approaches to paying workers (such as per weight of cane cut vs. per hour), and pace of sugarcane work differ in Kenya and Central America. Opportunities for water, rest, and shade (WRS) may differ as well, and WRS intervention studies in Central America appear to limit the impact of work on biomarkers of kidney function [25, 51].

Physically demanding workload was not found to be significantly associated with eGFR < 90 in multivariate models. Taken together, we found that neither work in the sugarcane industry, as a cane cutter, or in physically demanding occupations to be independent predictors of reduced eGFR in this population. These findings are in contrast to what Schlader et al. [28] reviewed, describing the potential mechanisms supporting how physical workload in the heat may lead to heat strain and dehydration, which then may increase one’s risk for acute kidney injury (AKI). Hansson et al. [26] built on this concept, hypothesizing that sugarcane cutters may have repeated exposures to hypoxia, fructose, uric acid, and the release of pro-inflammatory cytokines in tubuli due to physically demanding work in heat and rehydrating with sugary drinks. The results in the present study are consistent with arguments made by others that strenuous exertion in heat does not clearly lead to CKDu after recurrences of AKI [52]. Interestingly, in an occupational-based cohort study of sugarcane workers in Nicaragua, those who had the highest workload (sugarcane cutters) also had the greatest eGFR increase across the harvest season compared to those with lesser workloads [27].

Distinct from the issue of what sugarcane cutters do, the classification of job titles in population-based studies has not been standardized across CKDu studies. As a result, it is difficult to know what job duties workers have, how physically strenuous the work is, under what conditions, and for what duration. This limits our ability to interpret the observed prevalence of CKDu across studies and even within studies, across occupational groups. One study in India classified six categories of occupations, including homemaker, student, and unemployed; however, it was not clear the methods used to classify [53]. The current study sought to use a standardized set of occupational categories defined in ISCO-08 [39]. Nevertheless, this approach has its limitations. We asked participants about their “current” or “main” occupation, yet individuals may have multiple jobs at the same time or at different times of the year. The ISCO-08 was also not designed to ask follow-up questions about the working conditions, such as duration engaged in physically intense work. Improving CKDu surveillance should include improved methods to describe one’s occupation, including those who are not part of the formal workforce (e.g., homemakers).

The findings of this study are subject to several limitations. First, because the study design is cross-sectional reported exposures (e.g., sublocation, occupation, BMI, diet) and disease outcome (e.g., low eGFR) are measured at the same time for each participant, making it challenging to infer causality in the observed associations. Second, the generalizability of the study findings to other populations in East Africa and beyond is limited as only one sub-county of one county was studied. Third, the study enrolled only 12 participants who, at the time of the study, worked for the past three months cutting cane, limiting the statistical power of analyses of occupation and eGFR < 90. Additionally, the CKD-EPI equation has not been validated in African populations, which may lead to an underestimation of CKDu prevalence [44, 54–56]. However, this would not impact our assessment of associations between reduced eGFR and other factors, as the same equation was used for all participants. The study also had numerous strengths such as utilizing the standardized DEGREE protocol to estimate the distribution of eGFR in a population, a high enrollment rate (90.0%), and a representative population sample by using random selection of villages that included participants from both urban and rural settings.

## Conclusion

We found that in one sugarcane producing region of Western Kenya, CKDu prevalence was low, and that HIV was common among those with reduced eGFR. Thus, future studies of CKDu should account for HIV as a known cause of reduced renal function. If physically demanding work in a hot climate were the sole cause of CKDu, one might expect a high CKDu prevalence in the study area, given the equatorial setting in which sugarcane is grown and cut; however, this was not observed. The current findings suggest that in CKDu-endemic regions, heat stress and physically demanding work may interact with other exposures to put populations at risk for CKDu.

## Supplementary Information

**Additional file 1.**

## Data Availability

The datasets used and/or analyzed during the current study are available from the corresponding author on reasonable request.

## Abbreviations

CKD: Chronic kidney disease
CKDu: Chronic kidney disease of undetermined causes
eGFR: Estimated glomerular filtration rate
DEGREE: The Disadvantaged Populations eGFR Epidemiology Study
DM: Diabetes mellitus
HTN: Hypertension
HP: Heavy proteinuria
HIV: Human immunodeficiency virus
BMI: Body Mass Index
EPI: Epidemiology
ISCO-08: International Standard Classification of Occupations 2008
AKI: Acute kidney injury
WRS: Water, rest, shade
ART: Antiretroviral therapy
OR: Odds ratio
Pctl: Percentile
KEMRI: Kenya Medical Research Institute
IRB: Institutional Review Board
SAS: Statistical Analysis Software
CINAC: Chronic interstitial nephritis in agricultural communities
RPM: Revolutions per minute
